# Controlling for genetic identity of varieties, pollen contamination and stigma receptivity is essential to characterize the self‐incompatibility system of *Olea europaea* L.

**DOI:** 10.1111/eva.12498

**Published:** 2017-06-29

**Authors:** Pierre Saumitou‐Laprade, Philippe Vernet, Xavier Vekemans, Vincent Castric, Gianni Barcaccia, Bouchaïb Khadari, Luciana Baldoni

**Affiliations:** ^1^ CNRS UMR 8198 Evo‐Eco‐Paleo Université de Lille ‐ Sciences et Technologies Villeneuve d'Ascq France; ^2^ Laboratory of Genomics and Plant Breeding DAFNAE ‐ University of Padova Legnaro PD Italy; ^3^ UMR 1334 Amélioration Génétique et Adaptation des Plantes (AGAP) INRA/CBNMed Montpellier France; ^4^ UMR 1334 AGAP Montpellier SupAgro Montpellier France; ^5^ CNR Institute of Biosciences and Bioresources Perugia Italy

**Keywords:** diallelic self‐incompatibility system, *Olea europaea* L., Oleaceae, paternity analysis, plant mating systems, symmetry in reciprocal crosses

## Abstract

Bervillé et al. express concern about the existence of the diallelic self‐incompatibility (DSI) system in *Olea europaea*, mainly because our model does not account for results from previous studies from their group that claimed to have documented asymmetry of the incompatibility response in reciprocal crosses. In this answer to their comment, we present original results based on reciprocal stigma tests that contradict conclusions from these studies. We show that, in our hands, not a single case of asymmetry was confirmed, endorsing that symmetry of incompatibility reactions seems to be the rule in Olive. We discuss three important aspects that were not taken into account in the studies cited in their comments and that can explain the discrepancy: (i) the vast uncertainty around the actual genetic identity of vernacular varieties, (ii) the risk of massive contamination associated with the pollination protocols that they used and (iii) the importance of checking for stigma receptivity in controlled crosses. These studies were thus poorly genetically controlled, and we stand by our original conclusion that Olive tree exhibits DSI.

## 1 INTRODUCTION

Olive has been the iconic tree of the Mediterranean area due to its economical, ecological, cultural, and social importance over an extended period of human history, and a large number of studies have attempted to characterize its mating system. While no consensus model has emerged so far in the literature, some studies performed by the same group of authors (Breton & Bervillé, 2012; Breton et al., 2014; Farinelli, Breton, Famiani, & Bervillé, 2015; Koubouris, Breton, Metzidakis, & Vasilakakis, 2014) claimed to have identified at least six self‐incompatibility alleles, and asymmetrical crosses indicative of a classical sporophytic self‐incompatibility system. Our recent results (Saumitou‐Laprade et al., 2017) contradict several of these previously published conclusions and indicate that Olive rather shares the unusual diallelic self‐incompatibility (DSI) system previously discovered in *Phillyrea angustifolia*. Hence, according to those results, in Olive, only two incompatibility groups or genotypes do exist, with all individuals of a given group incompatible with each other and fully compatible with all individuals of the other group. As suggested in Saumitou‐Laprade et al. (2017), this discrepancy is probably due to several shortcomings in previously published analysis that lacked proper genetic control. In our view, the Comment to the Editor by Breton et al. (2017) fails to take into account specific challenges associated with the genetic analysis of the particular biological material represented by Olive trees. Besides several inaccurate statements in their comment, we outline below three major sources of uncertainty in the studies cited by Breton et al. (2017) that prevent conclusive evidence to be drawn about the rejection of our model for self‐incompatibility system of the Olive tree.

The first difficulty arises when comparing studies performed with plant material that is only referenced by variety's vernacular names. Indeed, a major conclusion of our work (Saumitou‐Laprade et al., 2017) was that it is essential to identify varieties by their reference genotype based on molecular markers rather than by their vernacular name (El Bakkali et al., 2013; Haouane et al., 2011; Trujillo et al., 2014), as this is associated with considerable confusion. Breton et al. (2017) contend that our results are inconsistent with their own studies. This is indeed true. To understand the origin of this discrepancy, we analyzed 66 trees from the olive collection cited in Saumitou‐Laprade et al.(2017), which includes *pro parte* some of the varieties cited in the Breton et al. (2017) comment (Breton et al., 2014; Farinelli et al., 2015). From these 66 individuals, we identified a total of 61 different genotypes using microsatellite markers and then matched these genotypes in the worldwide Olive World Germplasm Bank of INRA Marrakech assessed with the same markers. This simple analysis revealed no less than 14 cases where the genotypes associated with a given variety name were different among collections, representing a major discrepancy that demonstrates the unreliability of vernacular names. Hence, previous studies including those published by the Bervillé et al. group were based on poorly identified varieties, which is likely to have generated considerable uncertainty in the results. We believe that it will now be important for the community working on Olive trees to generate a public database of trees whose genetic identity has been ascertained by a common set of molecular markers, ideally also including their position in a reference orchard and a voucher DNA sample that could be exchanged among users. The *Arabidopsis thaliana* community has, for the same reasons, also recently launched a similar initiative (Bergelson, Buckler, Ecker, Nordborg, & Weigel, 2016).

The second challenge is taking into account the risk of pollen contamination when performing controlled crosses in the Olive. A careful analysis of the methods typically used to perform controlled crosses in Olive reveals that, except in case of self‐pollination in which the flowers remain protected during the whole process, the risk of contamination by pollen is indeed very high as Olive pollen is mostly wind dispersed. First, the twigs containing flowers to be pollinated are typically protected by a single bag in most studies. This protecting bag is opened at full blooming in the orchard to introduce pollen from fathers to be tested, either with a branch collected on the pollen donor tree or with a pencil. Massive contamination was demonstrated in *Olea europaea* in crosses following such a protocol (de la Rosa, James, & Tobutt, 2004) with as many as 96 of 149 (64%) of progenies whose expected father could be genetically excluded, and therefore resulting from pollen contamination. Similarly in *Phillyrea angustifolia*, a wild relative of Olive (Saumitou‐Laprade et al., 2010), the paternity analysis of progenies produced by handmade, apparently controlled, crosses following a similar protocol, revealed more than 50% of progenies produced by contaminant pollen (unpublished data). Using a more carefully controlled pollination protocol (Billiard et al., 2015; Saumitou‐Laprade et al., 2017), we showed that contamination could be decreased down to 1.7% (over 1,048 progenies tested, unpublished data). As we explained in Saumitou‐Laprade et al. (2017), we strongly believe that any conclusion based on a protocol that entails such a massive level of contamination should be treated with caution, if not entirely disregarded, if it is not associated with molecular tests of paternity designed to exclude contaminated seeds.

Finally, according to the Breton et al. (2017) comment, the main objection against the existence of DSI in Olive remains its inability to explain the asymmetry reported by Breton et al. (2014) and other authors (Farinelli, Boco, & Tombesi, 2006; Farinelli et al., 2015; Moutier, 2006; Spinardi & Bassi, 2012; Villemur, Musho, Delmas, Maamar, & Ouksili, 1984) in studies based on measurement of fruit set following reciprocal pollination between pairs of varieties. Because the experimental protocol applied to assess the DSI in Olive (Saumitou‐Laprade et al., 2017) was not designed to detect asymmetry in reciprocal crosses, we here present original results from reciprocal stigma tests performed with pairwise varieties for which asymmetry was published (Breton et al., 2014), strongly suggesting symmetrical instead of asymmetrical relationships. We also present results from diallelic crossing experiments performed with eight different varieties which could explain why so many symmetrical crosses between compatible varieties have been interpreted as asymmetrical.

## 2 PLANT MATERIAL AND METHODS TO ASSESS ASYMMETRY IN RECIPROCAL CROSSES

We worked with some varieties cited in Breton et al. (2014). We followed the protocol described in Saumitou‐Laprade et al. (2017): Stigma and pollen were collected on each individual tree minimizing the risk of pollen contamination, each individual tree was phenotyped for SI and was genotyped by 15 polymorphic microsatellite marker loci (Baldoni et al., 2009; El Bakkali et al., 2013). Therefore, we provide for each individual: a SI phenotype, a physical position in the orchard, a genotype corresponding to a specific combination of alleles at 15 polymorphic SSR loci (see Table S1 in Saumitou‐Laprade et al. (2017) for the 16 genotypes shared with the previous study, and Table S1 in the present study for Oit46).

**Figure 1:**
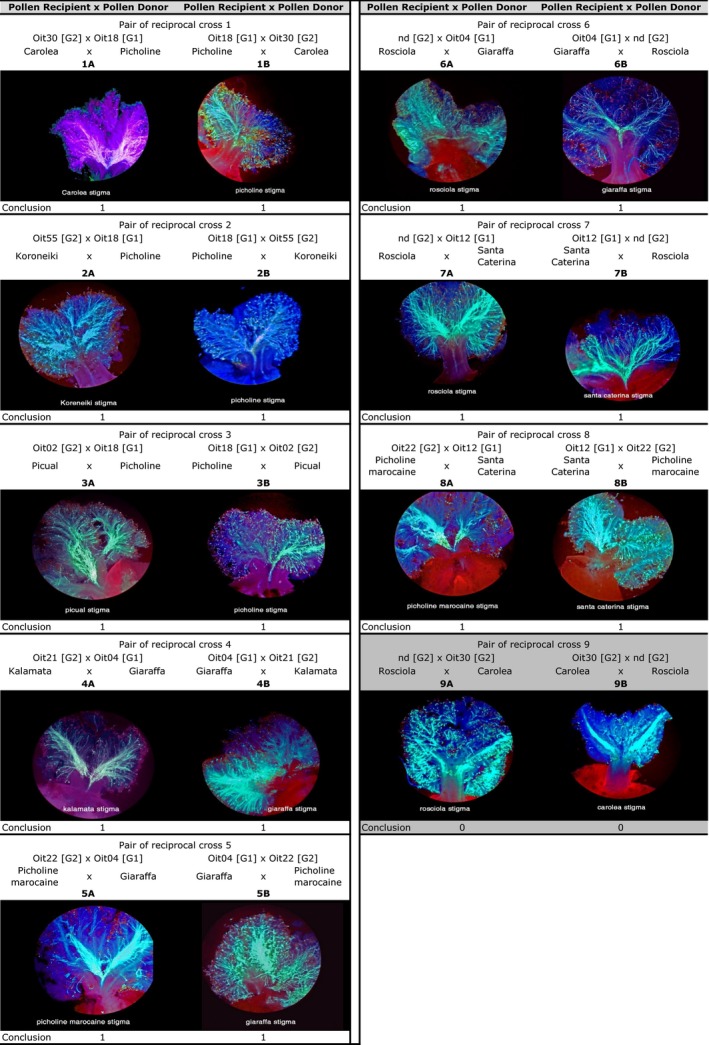
Reciprocal stigma tests in nine pairs of crosses performed with nine different Olive varieties previously phenotyped for SI group ([G1] and [G2], respectively) using stigma test defined in Saumitou‐Laprade et al. (2017). Pairs 1–8 correspond to compatible crosses (conclusion = 1) among varieties belonging to two different SI groups; pair 9 corresponds to incompatible cross (conclusion = 0) among varieties belonging to the same group. Phenotyped trees are labeled according to their reference genotype (Saumitou‐Laprade et al., 2017) and their variety name in the studied orchard. nd: not defined

In June 2013 and 2014, 17 different genotypes were chosen in the orchard and assigned to one of the two SI groups using stigma tests, as described in Saumitou‐Laprade et al. (2017). In a first experiment, nine genotypes were selected in order to replicate nine pairwise compatibility tests between varieties for which asymmetry has been reported in Breton et al. (2014). Reciprocal cross‐compatibility was assessed using stigma tests in pairwise tests (see Table 1 and Figure 1) following the protocol and criteria of Saumitou‐Laprade et al. (2017). In a second experiment, a multiple reciprocal stigma test involving four [G1] and four [G2] individual genotypes (Table 2) was conducted according to a diallelic design. Indeed, each individual previously assigned to one of the two SI groups using the two pairs of testers defined for the screening of a large collection of Olive trees (Saumitou‐Laprade et al., 2017) was used as pollen donor and pollen recipient in reciprocal crosses (including selfing controls). Note that three of the genotypes used in the multiple reciprocal stigma tests (namely Oit26, Oit15, and Oit65) correspond to tester genotypes in Saumitou‐Laprade et al. (2017). In order to allow some comparison between our results and those previously obtained by Berville et al., we decided to include in our figure and tables the labels reported in Saumitou‐Laprade et al. (2017) and their associated vernacular names (but keep in mind the uncertainty expressed in introduction). For one tree, referenced Oit46 and reported in the orchard under the “Grossane” variety name (Table S1), we used 10‐days‐old stigma. Stigma were protected from contaminant pollen by double bagging and transferred to the laboratory in bags still closed 10 days after the first flower opened on the tree, harvested from twigs under laboratory conditions, maintained 24 hr in petri dishes containing a Brewbaker and Kwack medium (Vernet et al., 2016) and pollinated.

**Table 1:** Results of reciprocal stigma tests performed between pairs of Olive varieties in which, according to Breton et al. (2014), asymmetry is either predicted by the model with six S alleles showing dominance relationships in pollen or deduced from fruit setting. In the nine reciprocal stigma tests, symmetry is observed and asymmetry rejected. Ref: reference of individual tree, [SI]: self‐incompatibility phenotype, nd: not determined

Pollen recipient			Pollen donor			Conclusions				
						In Breton et al. (2014)			In the present study	
Variety Name	Ref^a^	[SI]^b^	Variety Name	Ref^a^	[SI]^b^	Success of cross^c^ in fig. 2	Scheme in fig. 2 *expected genotype for SI*	Success of cross^d^ in Tab. 4	Pollen tubes growth	Photographs in Figure 1
Carolea	Oit30	G2	Picholine	Oit18	G1	0	B:*R3R4* × *R1R3*	1^e^	**1**	1A
Picholine	Oit18	G1	Carolea	Oit30	G2	1	B: *R1R3 *×* R3R4*	1	**1**	1B
Koroneiki	Oit55	G2	Picholine	Oit18	G1	0	C: *R3R4 *×* R1R3*	np	**1**	2A
Picholine	Oit18	G1	Koroneiki	Oit55	G2	1	C: *R1R3 *×* R3R4*	np	**1**	2B
Picual	Oit02	G2	Picholine	Oit18	G1	0	B: *R3R4 *×* R1R3*	np	**1**	3A
Picholine	Oit18	G1	Picual	Oit02	G2	1	B: *R1R3 *×* R3R4*	np	**1**	3B
Kalamata	Oit21	G2	Giaraffa	Oit04	G1	1	E: *R2R4 *×* R2R3*	1	**1**	4A
Giaraffa	Oit04	G1	Kalamata	Oit21	G2	0	E: *R2R3 *×* R2R4*	0	**1**	4B
Picholine Marocaine	Oit22	G2	Giaraffa	Oit04	G1	1	E: *R2R4 *×* R2R3*	np	**1**	5A
Giaraffa	Oit04	G1	Picholine Marocaine	Oit22	G2	0	E: *R2R3 *×* R2R4*	np	**1**	5B
Rosciola	nd	G2	Giaraffa	Oit04	G1	1	D: *R3R5 *×* R2R3*	np	**1**	6A
Giaraffa	Oit04	G1	Rosciola	nd	G2	0	D: *R2R3 *×* R3R5*	np	**1**	6B
Rosciola	nd	G2	Santa Caterina	Oit12	G1	1	D: *R3R5 *×* R2R3*	np	**1**	7A
Santa Caterina	Oit12	G1	Rosciola	nd	G2	0	D: *R2R3 *×* R3R5*	np	**1**	7B
Picholine Marocaine	Oit22	G2	Santa Caterina	Oit12	G1	1	E: *R2R4 *×* R2R3*	np	**1**	8A
Santa Caterina	Oit12	G1	Picholine Marocaine	Oit22	G2	0	E: *R2R3 *×* R2R4*	np	**1**	8B
Rosciola	nd	G2	Carolea	Oit30	G2	1	F: *R3R5 *×* R3R4*	np	**0**	9A
Carolea	Oit30	G2	Rosciola	nd	G2	0	F: *R3R4 *×* R3R5*	np	**0**	9B

a Reference of the tree used for phenotyping: its position in orchard and its genotype with 15 SSR markers are presented in Table S1 (Saumitou‐Laprade et al., 2017).

b Incompatibility group determined using stigma test and presented in Saumitou‐Laprade et al. (2017).

c In Breton et al. (2014), cross‐compatibility and incompatibility predicted between pairs of Olive varieties by the model with dominance relationships.

d In Breton et al. (2014), “successes of crosses are designated (1) at threshold over or equal to 0.8 for mean of crosses, below it was designated (0) which means the cross has failed, but it was expected (1)”; np: cross not performed.

e Discrepancy detected between predicted (see fig. 2) and observed (see table 4) compatibility/incompatibility relationships among varieties in Breton et al. (2014).

**Table 2:** Multiple reciprocal stigma tests performed between eight different Olive varieties in a diallelic scheme. Each individual was used as pollen donor and pollen recipient in reciprocal crosses (including selfing with itself). Stigma tests were performed and analyzed for compatibility/incompatibility conclusions according to Saumitou‐Laprade et al. (2017). 0: absence of pollen tube or presence of only short pollen tubes never reaching the style interpreted as incompatibility; 1: occurrence of several pollen tubes converging through the stigmatic tissue toward the style until the base of the stigma and entrance of the style interpreted as compatibility between parents. In grey shading results in case of self‐pollination

	Variety Name in Orchard	Ref^a^	[SI]^b^	Pollen donor							
				Arbequina	Dolce Agogia	Frantoio	Grossane	Leccino	Maurino	Moraiolo	Nostrale di Rigali
				Oit26	Oit15	Oit25	Oit46	Oit65	Oit17	Oit24	Oit58
				G1	G2	G1	G2	G1	G2	G1	G2
Pollen recipient	Arbequina	Oit26	G1	0	1	0	1	0	1	0	1
	Dolce_Agogia	Oit15	G2	1	0	1	0	1	0	1	0
	Frantoio	Oit25	G1	0	1	0	1	0	1	0	1
	Grossane	Oit46	G2	0 c	0	0 c	0	0 c	0	0 c	0
	Leccino	Oit65	G1	0	1	0	1	0	1	0	1
	Maurino	Oit17	G2	1	0	1	0	1	0	1	0
	Moraiolo	Oit24	G1	0	1	0	1	0	1	0	1
	Nostrale di Rigali	Oit58	G2	1	0	1	0	1	0	1	0

a Reference of the tree used for phenotyping: its position in orchard and its genotype with 15 SSR markers are presented in Table S1 (Saumitou‐Laprade et al., 2017).

b Incompatibility group determined using stigma test and presented in Saumitou‐Laprade et al. (2017).

c In red, dicrepancy detected between predicted and observed compatibility/incompatibility relationships among varieties.

## 3 RESULTS AND DISCUSSION

### 3.1 Symmetrical rather than asymmetrical incompatibility reactions in Olive

In eight cases, we observed pollen tubes converging through the stigmatic tissue toward the style until the base of the stigma and entrance of the transmitting tissue of the style, indicating perfect compatibility between parents of the crosses (see Table 1 and Figure 1, panels 1–8). In all reciprocal crosses, compatibility was observed in both directions of the reciprocal crosses. In the last cross, we observed only short pollen tubes that did not reach the style (see Table 1 and Figure 1, panel 9A) or the absence of pollen tubes growing within the stigma (Figure 1, panel 9B). These figures, typical for incompatibility reactions, were observed in the two directions of the reciprocal cross, thus demonstrating symmetrical incompatibility of the two parents. The compatibility/incompatibility relationships detected are all in agreement with the SI group assignment performed previously with stigma tests (see Table S1 in Saumitou‐Laprade et al. (2017)) or during the current study (Table 1) for “Kalamata” and “Rosciola,” hence demonstrating perfect reproducibility and full reliability of our results. Specifically, the genotypes belonging to the G1 SI group are reciprocally compatible with genotypes belonging to G2, and the genotypes belonging to the same SI groups are reciprocally incompatible. Therefore, in our experiments, any case of asymmetry was documented, suggesting that symmetry of incompatibility reactions appears to be the rule in Olive, as predicted in our model of diallelic SI. Why then did previously studies published by Bervillé et al. conclude to the contrary? Because of the pollination protocol used, and because none of the studies cited by Breton et al. (2014) were accompanied by paternity analyses, pollen contamination may have produced unreliable results. Nevertheless, it is worth mentioning that pollen contamination can only explain “false positive” errors: that is, seeds produced by means of crosses that should otherwise be incompatible (for instance, the seeds expected on “Rosciola” when pollinated by “Carolea”: See fig. 2, scheme F in Breton et al. (2014)).

To explain the absence of seeds produced by one of the two compatible parents in the eight additional crosses, *that is,* “false‐negative” errors, we analyzed the diallelic scheme among eight different Olive varieties (Table 2). Among the 64 stigma tests performed and analyzed for compatibility/incompatibility conclusions, 60 provided the expected results according to the SI phenotypes of the parental genotypes. The four discrepancies were observed when the reference Oit46 (reported with the variety name “Grossane” in the orchard) was used as a mother. Interestingly, stigma from the same genotype provided the expected compatibility result with Oit15 in the test for SI group assignment performed 10 days before the multiple reciprocal stigma tests. We observed an absence of pollen tube germination on the 10‐day‐old stigma from the Oit46 genotype with the four compatible genotypes (Oit26, Oit25, Oit65, and Oit24), which can be interpreted as a loss of receptivity of the flowers collected on this twig. Hence, stigma probably lost receptivity 10 days after the first flower opened on the tree. This finding is in agreement with reported values for the effective pollination periods (EPP) determined in Olive orchards from California and Spain (Cuevas, Pinillos, & Polito, 2009), although these values varied across years and varieties. It is worth noting that in the present study, all 10‐day‐old flowers we collected on the Oit46 genotype were looking very fine as morphological appearance and no sign of senescence was detected in the stigma. Such a lack of receptivity could explain why previous studies reported asymmetry in reciprocal crosses. Indeed, reciprocal crosses performed by transferring one branch of the pollen donor into the bag protecting flowers of the pollen recipient actually require pollen release by the two partners. Because anther dehiscence is rarely synchronized among partners (depending on their genotype and/or position in the orchard), receptivity of the early‐flowering partner may be lost when dehiscence begins in the late one. Pollen from the early‐flowering partner may be still alive and able to fertilize receptive flowers from late‐flowering partner, whereas stigma from the former may have lost receptivity and cannot be fertilized by pollen from the later. We suggest that asymmetries reported in literature may correspond to false‐negative results between compatible mates whose periods of blooming were not sufficiently synchronized.

## 4 CONCLUSIONS

The mating system of the Olive tree has remained a controversial issue in the literature, but many of the previously published studies have been based on a poorly genetically controlled experimental design. Given 1) the vast uncertainty around the genetic identity of vernacular varieties, 2) the massive risk of contamination associated with commonly used pollination protocols and 3) the importance of checking for stigma receptivity in controlled crosses [all important features that were not adequately taken into account in the studies cited by the Breton et al.(2017) comment], we are confident that the time is ripe for new standards to be set in the scientific community. We can only encourage authors of this comment as well as any other researchers having doubts about the actual existence of DSI and the absence of asymmetrical incompatibility reactions in Olive trees, to carefully assess reproducibility of the output data of their experimental crosses, to control for pollen contamination with paternity analyses and to use positive pollination controls of stigma receptivity. We believe that accurate tests of our proposed model of diallelic SI in Olive need to be performed by other teams in a larger set of genotypes, in order to confirm the generality of our observation, but they should take into account our suggestions to avoid misleading results. Other scientific communities have strongly benefited from directly ascertaining genetic relationships, eventually leading to drastic changes of their paradigmatic interpretation (Bergelson et al., 2016; Griffith, Owens, & Thuman, 2002). We believe that would be now a good time for the Olive tree research community to join this general movement.

## 5 FEW REMARKS IN RESPONSE TO OTHER CRITICISMS OF THE BRETON ET AL.(2017) COMMENTS

1. *Mistake in the legend of table *2 *in Saumitou‐Laprade* et al.*,* *2017* *)*: The cross described in Table 2 is correctly described in Material and Methods section, but there is a mistake in the legend title of the table: It is written (Oit64 × Oit27) instead of (Oit27 × Oit15). We apologize for this mistake and thank the authors of the comment for their remark. Nevertheless, this error does not change the conclusions of the genetic analysis of the cross which shows the 1:1 segregation of progenies that are all self‐incompatible and equally distributed among the two SI groups (and not a “segregation for self‐fertility” as written in the comment).
2. *Arguments in favor of the sporophytic nature of the SI in O. europaea*. We underlined in Saumitou‐Laprade et al. (2017) that none of the arguments presented in literature was decisive and we presented two arguments based on original results we obtained. The first argument that establishes the sporophytic nature of the self‐incompatibility system refers to the 1:1 proportion of the two parental SI groups in the controlled‐cross progeny that excludes the possibility of gametophytic control of self‐incompatibility (GSI) (Bateman, 1952). The second argument refers to the requirement of GSI, to be functional, of a minimum of three S alleles (with strict codominance between S alleles in the pistil to avoid compatibility of heterozygous individuals), and that defines a minimum of three incompatibility groups (Hiscock & McInnis, 2003). The two groups observed in *O. europaea* can be explained, only by a sporophytic diallelic SI system.
3. *Arguments about the risk of mismatch in assigning the correct father in paternity analysis based on DNA isolated from embryo*. Most Oleaceae species (including *O. europaea*,* P. angustifolia*,* Fraxinus ornus,* and *F. excelsior*) present more than one ovule in their gynoecium and can potentially produce more than one embryo in a single fruit. Nevertheless, this has never been reported to be a problem in the different studies having tested paternity using DNA isolated from embryos in *O. europaea* (Mookerjee et al., 2005; Diaz 2006, Besnard 2009, Marchese 2016, Saumitou‐Laprade et al., 2017), in *P. angustifolia* (Vassiliadis et al. 2002), in *F. ornus* (Verdu et al. 2006), or in *F. excelsior* (Bochenek 2011). In fact, multiple embryos are the exception in *O. europaea* (a single embryo is the rule), and embryos from a single fruit are very easily separated and treated as two different samples for DNA extraction.

## DATA AND MATERIAL SHARING

All relevant data are within the paper and its Supporting Information files.
