# Supplementary material for: Controlling for genetic identity of varieties, pollen contamination and stigma receptivity is essential to characterize the self‐incompatibility system of Olea europaea L
Source: Evol Appl. 2017 Jun 29;10(9):860–6. doi: 10.1111/eva.12498 (PMC5680419; doi:10.1111/eva.12498)
Supplement: Supplementary file 1 [file EVA-10-860-s001.pdf]

Table S1. Genotype of the Oit46 Olive varieties of the Perugia collection (PER) (Italy) with 15 polymorphic SSR markers. The tree was tested for compatibility with G1 and G2 testers: 0 indicates incompatibility was observed, 1 indicates compatibility was observed. Each tree was assigned to G1 or G2 SI group. Assignment was repeated with different pairs of testers.

Collection / Orchard	Position in collection / orchard	Reference DNA sample code	compatibility with G1	compatibility with G2	compatibility with G1+G2	SI Phenotype	repetitions of stigma test	Self-Incompatibility	Self Compatibility	Gene pool	DCA01	DCA01	DCA03	DCA03	DCA04	DCA04	DCA05	DCA05	DCA08	DCA08	DCA09	DCA09	DCA11	DCA11	DCA14	DCA14	DCA15	DCA15	DCA18	DCA18	EMO03	EMO03	EMO90	EMO90	GAPU59	GAPU59	GAPU71B	GAPU71B	UDO36	UDO36
PER	M-40	Oit46	1	0	0	G2	2	1	0	Central	203	211	236	250	130	161	190	202	135	137	159	169	145	177	176	186	242	242	174	176	209	212	183	189	210	210	126	139	140	142
OWGB	I-23-34	Oc1414																																						
